# Adaptive Traffic Signal Control: Game-Theoretic Decentralized vs. Centralized Perimeter Control

**DOI:** 10.3390/s21010274

**Published:** 2021-01-03

**Authors:** Maha Elouni, Hossam M. Abdelghaffar, Hesham A. Rakha

**Affiliations:** 1 Center for Sustainable Mobility, Virginia Tech Transportation Institute, Virginia Tech, Blacksburg, VA 24061, USA; emaha@vt.edu (M.E.); or hossamvt@vt.edu (H.M.A.); 2 Department of Computer Engineering and Systems, Engineering Faculty, Mansoura University, Mansoura 35516, Egypt; 3 Charles E. Via, Jr. Department of Civil and Environmental Engineering, Virginia Tech, Blacksburg, VA 24061, USA

**Keywords:** perimeter control, NFD, adaptive control, game theory, DNB

## Abstract

This paper compares the operation of a decentralized Nash bargaining traffic signal controller (DNB) to the operation of state-of-the-art adaptive and gating traffic signal control. Perimeter control (gating), based on the network fundamental diagram (NFD), was applied on the borders of a protected urban network (PN) to prevent and/or disperse traffic congestion. The operation of gating control and local adaptive controllers was compared to the operation of the developed DNB traffic signal controller. The controllers were implemented and their performance assessed on a grid network in the INTEGRATION microscopic simulation software. The results show that the DNB controller, although not designed to solve perimeter control problems, successfully prevents congestion from building inside the PN and improves the performance of the entire network. Specifically, the DNB controller outperforms both gating and non-gating controllers, with reductions in the average travel time ranging between 21% and 41%, total delay ranging between 40% and 55%, and emission levels/fuel consumption ranging between 12% and 20%. The results demonstrate statistically significant benefits of using the developed DNB controller over other state-of-the-art centralized and decentralized gating/adaptive traffic signal controllers.

## 1. Introduction

Traffic growth within urban roadway systems, in combination with limited available capacity, affects traveler mobility and air quality and impacts public health. Vehicles burn fuel at a higher rate in congestion, releasing emissions that contribute to air pollution, which is related to global warming [1]. These conditions can be improved by reducing congestion. Traffic signal controllers are one means of reducing congestion if traffic signals are adjusted properly, changing signal control variables such as phase sequences, cycle lengths, phase splits, and offsets. These controls can introduce significant improvements in traffic variables such as delays, travel times, and vehicle emissions.

**Figure 1:**
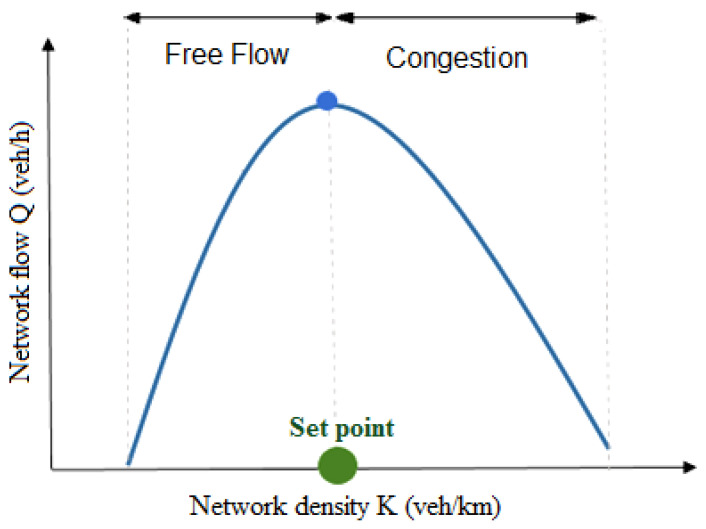
Network fundamental diagram.

One pertinent example of adaptive signal controllers for urban networks is perimeter flow control, or gating. The idea behind gating is to limit the flow entering a protected network (PN) in order to prevent congestion from occurring and dispersing congestion after it occurs. This method is based on the NFD [2,3,4], which identifies the network density at capacity (or set point) at which congestion starts to build up (Figure 1). The perimeter controller will then try to maintain the network density around the density at capacity, where the flow is maximal.

NFD based perimeter controllers assume a well-defined NFD [5]. The congestion inside the PN has to be homogeneous [3], meaning that the variance in network link densities must be minimal. In real life, this is not always achievable, especially in congested city centers where some links are very congested while others are not. Furthermore, the identification of the location and the number of the border signals where the gating is applied is crucial and could degrade the controller’s performance. Finally, gated links should be long enough to hold the long queues formed as part of the gating control. A previously developed decentralized Nash bargaining (DNB) controller [6,7] is an adaptive game-theoretic controller that was proven to perform well on different networks. Applying the DNB controller to resolve network congestion is advantageous since it does not require the identification of an NFD and its associated challenges, and given that the system is decentralized, it is scalable and thus easy to apply to large-scale networks.

In this paper, the developed DNB controller is compared to the operation of an optimum fixed-time coordinated plan (FP), a centralized adaptive phase split controller (PS), and a decentralized adaptive phase split and cycle length controller (PSC) [8]. Feedback gating control (at the PN border traffic signals) is combined with local traffic signals (at all other traffic signals inside and outside the PN). Perimeter control was applied to alleviate the congestion whenever it occurs [5,9]. The performances of the combination of FP with gating (FPG), PS with gating (PSG), and PSC with gating (PSCG) are compared to the performances of the traffic signal controllers without gating (i.e., FP, PS, PSC) and to the recently developed DNB traffic signal controller. To assess the controllers’ performance, a replication of the grid network of downtown Washington, D.C., was modeled in the INTEGRATION microsimulator software [10,11]. The central area of the PN was homogeneously congested, having a well-defined NFD.

These controllers were implemented and evaluated in the INTEGRATION software, which uses the Rakha–Pasumarthy–Adjerid car-following model to replicate the longitudinal movement of vehicles [12]. Vehicle movement is constrained by a vehicle dynamic model described in [13]. In [14], the model estimates of vehicle delay were validated, whereas in [15], the vehicle stop estimates were described and validated. The lateral movement of a vehicle is modeled using lane-changing models described in [16]. The VT-Micro model [13,17] is used to estimate vehicle fuel consumption and emissions levels.

This paper is organized as follows. Section 2 describes the related work. Section 3 describes the mathematical model of the protected network and the design of the proportional integral gating controller. The developed DNB controller is presented in Section 4. Section 5 describes the experimental setup, the experimental results, and the statistical analysis on a grid network. Section 6 presents the paper’s conclusions.

## 2. Related Work

Traffic signal systems may be categorized as either fixed-time, actuated, or adaptive. Timings in fixed-time control are computed offline using historical data. These timings are fixed and do not adapt to real-time traffic conditions except in the case of traffic responsive control where plans are introduced when traffic conditions warrant their introduction. Actuated controllers use data from detectors, located at traffic signal intersection stop lines, to respond to changes in traffic demand. In adaptive controllers, timings are computed in real time using real-time data obtained from cameras, loop detectors, or predictive models [18]. The split cycle offset optimization tool (SCOOT) [19], the Sydney coordinated adaptive traffic system (SCATS) [20], optimization policies for adaptive control (OPAC) [21], and RHODES [22] are examples of traditional adaptive traffic signal controllers. Both SCATS and SCOOTS are centralized controllers, whereas OPAC and RHODES are distributed.

Back-pressure based methods are widely used as adaptive traffic signal controllers. Wongpiromsarn et al. [23] were the first who adapted the back-pressure method from routing in communication networks to traffic signal control. This method assigns green time for different phases based on the back-pressure coefficient calculated as the difference in traffic status between upstream and downstream links. A commonly used criterion is the queue length of each approach. A standardized queue length based on link capacity is also used [24]. Considering each lane group separately, a multi-commodity model for back-pressure was demonstrated to perform better than the original method [25]. Back-pressure methods may have an unordered phasing sequence where the phase having the longer queue length is served first, or a fixed and ordered phase sequence like in [26,27] to ensure accommodating all approaches.

Different adaptive traffic signal controllers are designed based on heuristic and intelligent approaches, such as fuzzy sets [28,29], reinforcement learning [30], neural networks (NN) [31,32], and genetic algorithms [33,34]. A detailed review of these methods can be found in [35]. Intelligent approaches are useful when unexpected problems with traffic conditions occur (like car accidents, inclement weather, etc). The genetic algorithm is best used for simple and static problems, as it is computationally expensive in large-scale networks. The same holds true for fuzzy controllers, which are suitable for isolated intersections [36]. NN is used in S-TRAC (system-wide traffic adaptive controller) to generate optimum instantaneous timings of the signal [31]. In [37], the NN used real time data as the input and created different traffic time plans as the output. Most of the proposed NN adaptive traffic signal controllers are distributed, meaning that traffic signal timings are updated at a single intersection without consideration of its implication on other traffic signals. NNs also do not adapt quickly to changes in traffic because of the continuous online learning.

Reinforcement learning (RL) is a machine learning approach that allows agents to interact with the environment, trying to learn the best behavior through interaction feedback. Feedback may be available immediately after the event or several steps later, which makes it more difficult to learn. A Q-learning method was applied by Abdulhai et al. [38] in a two-dimensional road network to a simple isolated two phase traffic signal. Q-learning was applied in [38,39], where each agent decides the phase split in simple networks. Coordination between agents was taken into consideration in El-Tantawy et al. [30]. Their approach was tested on a network with 59 intersections, which is considered a small network [40]. More research is needed to test the effectiveness of intelligence based approaches on larger more realistic networks composed of hundreds of traffic signals.

Interactive cooperation between intelligent agents is studied in game theory. Bazzan [41] applied game theory to traffic control, where each agent represents a traffic signal having different signal plans defined a priori. This method works better when traffic is stable. The theory of bargaining is related to cooperative games via the Nash bargaining (NB) concept where multiple players with different objectives reach a mutually agreeable solution [42]. We developed a traffic signal controller based on a decentralized NB (DNB) [6,7] and showed that it is effective at improving network performance and reducing congestion. The operation of conventional adaptive controllers is limited by minimum and maximum cycle lengths, green timings, and offsets and also requires a predefined phase sequence. In addition, some systems use hierarchies that can partially or fully centralize decisions and make them susceptible to failures. The proposed DNB controller is a decentralized adaptive traffic signal controller, with a flexible phasing sequence and cycle-free operation, using an NB game-theoretic framework.

Numerous real-time perimeter controllers have been developed based on control theory, including: a standard proportional-integral (PI) controller [5,43], a robust PI controller [44], a sliding mode controller [45], a model predictive controller (MPC) [46,47], and a linear quadratic (LQ) controller [48]. The literature [5,49,50] shows that the PI perimeter controller is simple and efficient at reducing traffic congestion in an urban network. The combination of perimeter control with traditional traffic responsive local controllers was studied in [51,52], with the results indicating that the combination benefited the network performance.

Controllers for the traffic signal can be classified as centralized or decentralized. Decentralized systems have numerous advantages over centralized systems because they are less computationally demanding, require only relevant information from adjacent intersections/controllers, are robust, scalable, economical to set up and operate, and do not require a reliable direct network of communication among central computers and local controls. In this paper, the operation of the developed DNB controller is compared to the operation of the state-of-the-art decentralized, centralized, and gating traffic signal controllers.

## 3. Proportional-Integral Gating Control

This section describes the mathematical model of the PN (Section 3.1) and the design of the PI gating controller (Section 3.2).

### 3.1. Network State Space Model

**Figure 2:**
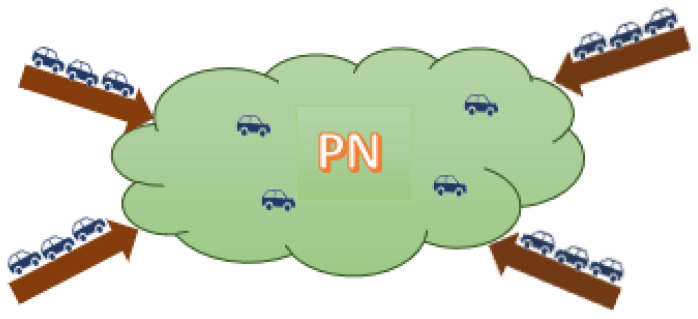
Protected network (PN).

This section presents the mathematical model of the PN. The NFD shown in Figure 1 presents an aggregated relationship between the density (K) and the flow (Q) in a protected (controlled) network. The objective is to control the vehicular input flow rate to the PN (shown in Figure 2) to maintain the density inside the PN around a specific set-point ($\bar{K}$) (i.e., to ensure that the PN does not enter the congested regime). The rest of this section derives the relation between the PN density and the input flow rate. It should be noted that an earlier paper demonstrated how the NFD can be constructed from probe data and the relationship between the network flow, *Q*, density, *K*, and space-mean speed, *V* [53].

The nonlinear relationship between the PN flow (Q) and the density (K) shown in Figure 1 is presented by Equation (1):

(1) Q(t)=G(K(t))

The NFD function (G(.)) is computed based on loop detector measurements of the average network density (*K*) and the average network flow (*Q*) inside the PN. The average network density *K* was computed using the following equation:

(2) $$K\left(n\right)=\sum_{z\in Z}K_{z}\left(n\right).L_{z}/\sum_{z\in Z}L_{z}$$

where *z* is a link index, *Z* is the set of measurement links, *n* is an index reflecting the time step, $L_{z}$ is the length of link *z*, $K_{z}\left(n\right)$ is the measured density on link *z* during time step *n*, and is calculated using Equation (3), where $l_{z}$ is the number of lanes on link *z*, ${k_{j}}_{z}$ is the jam density of *z*, and $o_{z}$ is the percentage of measured time-occupancy on link *z* during time step *n*.

(3) $$K_{z}\left(n\right)=l_{z}.{k_{j}}_{z}.o_{z}\left(n\right)/100$$

The flow (*Q*) inside the PN was calculated as shown in Equation (4), where $Q_{z}\left(n\right)$ represents the measured flow on link *z* during time step *n*.

(4) $$Q\left(n\right)=\sum_{z\in Z}Q_{z}\left(n\right).L_{z}/\sum_{z\in Z}L_{z}$$

The conservation equation for the vehicles inside the PN is shown in Equation (5), where *N* represents the number of vehicles in the PN, $q_{in}$ represents the PN input flow rate, $q_{out}$ represents the PN output flow rate, and $q_{d}$ represents the disturbance in the PN.

(5) $$N^{\prime }\left(t\right)=q_{in}\left(t\right)+q_{d}\left(t\right)-q_{out}\left(t\right)$$

Again, the objective is to control the input flow rate ($q_{in}\left(t\right)$) based on the PN density (K(t)) to maintain the density at a specific set-point ($\bar{K}$). Therefore, a relationship between $q_{in}\left(t\right)$ and K(t) must be established. First, the relationship between N(t) and K(t) is shown. Equation (6) shows the relationship between the measured density (*K*) from loop detectors and the real density ($K_{r}$), considering that the loop detectors may not be available in all PN links. That is represented by a correction factor (*R*), where 0<R≤1, and $\varepsilon_{1}$ represents the uncertainty (error) in the measured density.

(6) $$K_{r}\left(t\right)=K\left(t\right)/R+\varepsilon_{1}\left(t\right)$$

The relationship between *N* and $K_{r}$ is shown in the following equation (Equation (7)), where $L_{T}$ represents the sum of links’ lengths in the PN.

(7) $$K_{r}\left(t\right)=\frac{N(t)}{L_{T}}$$

Hence, from Equations (6) and (7), the relationship between *N* and *K* can be deduced (Equation (8)).

(8) $$N\left(t\right)=\frac{L_{T}}{R}K\left(t\right)+L_{T}\varepsilon_{1}\left(t\right)=\frac{L_{T}}{R}K\left(t\right)+\varepsilon_{2}\left(t\right)$$

Then, the relationship between $q_{out}\left(t\right)$ and K(t) is shown based on the NFD. Equation (9) shows the relationship between the measured (*Q*) and real ($Q_{r}$) flow in the PN, considering that the loop detectors may not be available in all the PN links. This is represented by a correction factor (*R*), where 0<R≤1, and $\varepsilon_{3}$ represents the uncertainty (error) in the measured flow.

(9) $$Q_{r}\left(t\right)=Q\left(t\right)/R+\varepsilon_{3}\left(t\right)$$

We assume that the exit flow rate ($q_{out}\left(t\right)$) is proportional to the real PN flow $Q_{r}\left(t\right)$ through Equation (10), where 0≤E≤1 [5].

(10) $$q_{out}\left(t\right)=EQ_{r}\left(t\right)$$

From Equations (1), (9), and (10), the relationship between $q_{out}\left(t\right)$ and K(t) is shown in Equation (11).

(11) $$\begin{array}{cc} \; & q_{out}\left(t\right)=E\left(G\left(K\left(t\right)\right)/R+\varepsilon_{3}\left(t\right)\right)\\ \; & \;=EG\left(K\left(t\right)\right)/R+E\varepsilon_{3}\left(t\right)\\ \; & \;=EG\left(K\left(t\right)\right)/R+\varepsilon_{4}\left(t\right) \end{array}$$

Substituting Equations (8) and (11) into Equation (5) yields the relationship between the rate of change in the density (*K*) in the PN and the input flow ($q_{in}$), where $\varepsilon =-\varepsilon_{4}\left(t\right)R/L_{T}$.

(12) $$\begin{array}{cc} \; & \frac{d}{dt}\left(L_{T}K\left(t\right)/R+\varepsilon_{2}\left(t\right)\right)=q_{in}\left(t\right)+q_{d}\left(t\right)\\ \; & \;-\left(EG\left(K\left(t\right)\right)/R+\varepsilon_{4}\left(t\right)\right)\\ \; & \frac{d}{dt}\left(K(t)\right)=\left(q_{in}\left(t\right)+q_{d}\left(t\right)-\left(G\left(K\left(t\right)\right)E/R\right)\right)R/L_{T}+\varepsilon \end{array}$$

The objective is to maintain the flow inside the PN at its maximum rate (i.e., the density inside the PN at a specific steady state point ($\bar{K}$)), and the controller is activated only if $K>0.85\bar{K}$, to avoid congestion. Therefore, we can linearize the NFD (G(K(t)) in Equation (12) at $\bar{K}$, as shown in Equation (13), where at steady state, ${\bar{q}}_{in}+{\bar{q}}_{d}={\bar{q}}_{out}$, and ${\bar{q}}_{out}=E{\bar{Q}}_{r}\left(t\right)=E/R\bar{Q}\left(t\right)$.

(13) $$G\left(K\left(t\right)\right)\approx \underset{\bar{Q}}{\underset{︸}{G(\bar{K})}}+\underset{{\bar{G}}^{\prime }}{\underset{︸}{\frac{dG(t)}{dK}{|}_{K=\bar{K}}}}\underset{K}{\underset{︸}{\left(K-\bar{K}\right)}}=\underset{{\bar{q}}_{out}.R/E}{\underset{︸}{\bar{Q}}}+{\bar{G}}^{\prime }K$$

Substituting Equation (13) into (12) yields:

(14) $$\frac{d}{dt}\left(K(t)\right)=\left(q_{in}\left(t\right)+q_{d}\left(t\right)-\left(\bar{q_{in}}+\bar{q_{d}}\right)-\frac{E}{R}{\bar{G}}^{\prime }K)\;\right)R/L_{T}+\varepsilon$$

Using the notation $x=x-\bar{x}$, where $\bar{x}$ is the steady state value, the linearized continuous time state equation is shown in Equation (15).

(15) $$\frac{d}{dt}\left(K(t)\right)=\left(q_{\mathrm{in}}\left(t\right)+q_{d}\left(t\right)-\frac{E}{R}{\bar{G}}^{\prime }K)\;\right)R/L_{T}+\varepsilon$$

The continuous time state space equation for the PN is shown in Equation (16), where $w\left(t\right)=q_{d}\left(t\right)R/L_{T}+\varepsilon$, $A=-E{\bar{G}}^{\prime }/L_{T}$, and $B=R/L_{T}$.

(16) $$K^{\prime }\left(t\right)=AK\left(t\right)+Bq_{\mathrm{in}}\left(t\right)+w\left(t\right)$$

The discrete state equation, assuming zero-order holds, is shown in Equation (17), where *n* represents the time step, $A_{d}=e^{AT}$, *T* is the sampling time, and $B_{d}=A^{-1}\left(A_{d}-1\right)B$.

(17) $$K\left[n+1\right]=A_{d}K\left[n\right]+B_{d}q_{\mathrm{in}}\left[n\right]+w\left[n\right]$$

The two model parameters $A_{d}$ and $B_{d}$ are calculated using a least-squares approximation (Equation (18)) of the measured data ($q_{{\mathrm{in}}_{m}},K_{m}$) near $\bar{K}$. Given that the error (ε) and the disturbance ($q_{d}$) are unknown and the model parameters $A_{d}$ and $B_{d}$ will be estimated from the measured data, we can assume that the noise (w[n]) will be incorporated in the least-squares approximation process and assume w[n]=0.

(18) $$\min_{A_{di},B_{di}}\sum_{i=1}^{I}{\left(K_{m}\left[i+1\right]-A_{di}K_{m}\left[i\right]-B_{di}q_{{\mathrm{in}}_{m}}\left[i\right]\right)}^{2}$$

The discrete PN transfer function between the output (K) and the input ($q_{\mathrm{in}}$) is shown in the following equation,

(19) $$PN\left(z\right)=\frac{K(z)}{q_{\mathrm{in}}\left(z\right)}=\frac{B_{d}}{z-A_{d}}\;$$

### 3.2. PI Controller

**Figure 3:**
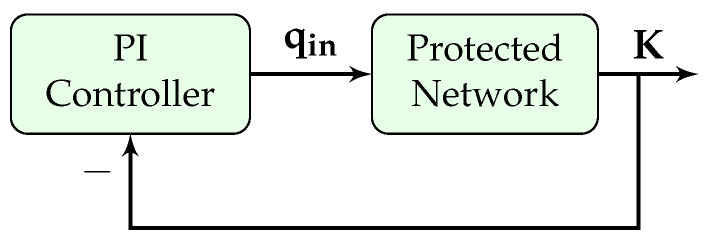
PI controller block diagram.

This section presents the design of the PI gating controller. The block diagram of the controller is presented in Figure 3, where the objective is to control the input flow rate ($q_{in}$) to keep the PN density (*K*) at a specific set-point ($\bar{K}$); the controller input flow ($q_{in}$) is distributed among the gated links.

The controller is activated as *K* reaches $0.85\;\bar{K}$, or deactivated if $K<0.85\;\bar{K}$. The following equation shows the error signal:

(20) $$e\left(t\right)=-K=\bar{K}-K$$

The PI controller equation is shown in Equation (21), where $K_{P}$ is the proportional gain and $K_{I}$ is the integral gain.

(21) $$q_{\mathrm{in}}\left(t\right)=K_{P}e\left(t\right)+K_{I}\int_{0}^{t}e\left(\tau \right)d\tau$$

The discrete time equation of the controller is shown in the following equation:

(22) $$q_{\mathrm{in}}\left(n\right)=q_{\mathrm{in}}\left(n-1\right)+K_{P}\left(e(n)-e(n-1)\right)+K_{I}e\left(n\right)$$

The discrete controller transfer function between the output ($q_{\mathrm{in}}$) and the input (*e*) is shown in the following equation:

(23) $$C\left(z\right)=\frac{q_{\mathrm{in}}\left(z\right)}{e(z)}=\frac{{(K}_{P}{+K}_{I})z-K_{P}}{z-1}$$

The overall closed loop transfer function of the block diagram shown in Figure 3 is shown in the following equation:

(24) $$\begin{array}{cc} \; & cl\left(z\right)=\frac{C(z).PN(z)}{1+C(z).PN(z)}\\ \; & \;=\frac{{(K}_{P}{+K}_{I}).\frac{z-\frac{K_{P}}{{(K}_{P}{+K}_{I})}}{z-1}.\frac{B_{d}}{z-A_{d}}}{1+{(K}_{P}{+K}_{I}).\frac{z-\frac{K_{P}}{{(K}_{P}{+K}_{I})}}{z-1}.\frac{B_{d}}{z-A_{d}}} \end{array}$$

Pole-zero cancellation and deadbeat control are used for tuning the PI controller (i.e., the assignment of $K_{P}$ and $K_{I}$ values) [54]. Following the pole-zero cancellation process, where the zero of the controller *C* (which is $\frac{K_{P}}{{(K}_{P}{+K}_{I})}$) is set equal to the pole of the PN (which is $A_{d}$), a relationship between the controller gains and $A_{d}$ can be shown in the following equation:

(25) $$A_{d}=\frac{K_{P}}{{(K}_{P}{+K}_{I})}$$

substituting Equation (25) into Equation (24), the closed loop transfer function becomes:

(26) $$cl\left(z\right)=\frac{{(K}_{P}{+K}_{I})\frac{B_{d}}{z-1}}{1+{(K}_{P}{+K}_{I})\frac{B_{d}}{z-1}}=\frac{{(K}_{P}{+K}_{I})B_{d}}{z-1+{(K}_{P}{+K}_{I})B_{d}}$$

Applying the fastest possible deadbeat control design, where the response of the system quickly reaches a zero error at the sampling instants, with $cl\left(z\right)=\frac{1}{z}$, a relationship between the controller gains and $B_{d}$ can be shown in the following equation:

(27) $${(K}_{P}{+K}_{I}).B_{d}=1$$

Solving Equations (25) and (27), the PI controller gains ($K_{P}\;\mathrm{and}\;K_{I}$) in terms of the model parameters ($A_{d}\;\mathrm{and}\;B_{d}$) are shown below:

(28) $$K_{P}=\frac{A_{d}}{B_{d}}\;K_{I}=\frac{1-A_{d}}{B_{d}}$$

## 4. DNB Traffic Signal Controller

In this section, a description of the application of the NB for two players (for illustration purposes only) at a single intersection is presented followed by a description of the decentralization of the controller over a network of traffic signals.

### 4.1. DNB Solution for Two Players

The bargaining problem is based on three basic elements: players, actions, and utilities (or rewards) [55,56]. The players in a bargaining situation cooperate and benefit by finding a mutual agreement. Bargaining between two players is presented in Table 1. The players in this study are traffic signal phases. Each player (phase), namely $P_{1}$ and $P_{2}$, has two possible actions $A_{1}$ (maintain) and $A_{2}$ (change). Maintain means that the signal state will stay the same (i.e., if it is green, it will stay green; if it is red, it will stay red). Change indicates that the signal state will change (i.e., if it is green, it will switch to yellow and then red; if it is red, it will become green) during the simulated time interval. The rewards of each player are u and v, respectively, as they take relevant actions. The utility function in this study is represented by the vehicles’ queue length at the traffic signal stop bar.

**Figure 4:**
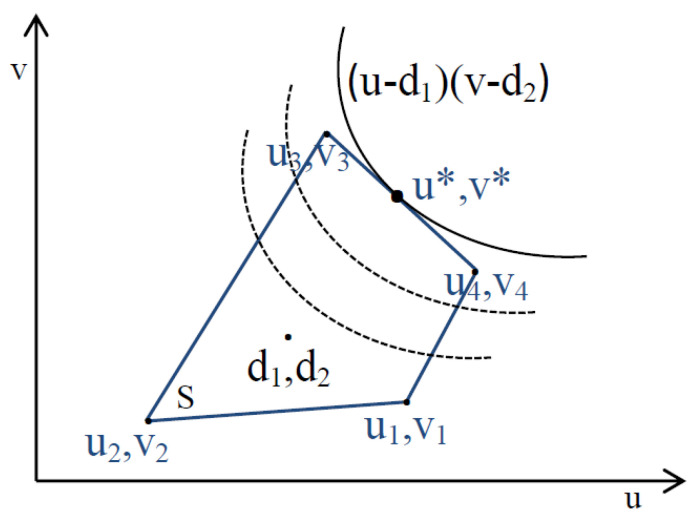
Utility region.

The space (S; Figure 4) is the set of all possible rewards for the two players. The disagreement point $d=(d_{1},d_{2})$ is the minimum utilities that the players want to achieve (i.e., maximum queue length). This point is a benchmark selected based on the fact that each player wants to maximize his/her benefit. Subsequently, a bargaining problem is defined as the pair (S,d) where $S\in R^{2}$ and d∈S such that S is a convex and compact set, and there exists some s∈S such that s>d [7].

Nash’s theorem proved the existence and uniqueness of the bargaining problem solution based on four axioms (Pareto efficiency, symmetry, invariance to equivalent utility representation, and independence of irrelevant alternatives). This solution is the pair of utilities $\left(u^{*},v^{*}\right)$ that solves the optimization problem presented in Equation (29):

(29) $$\begin{array}{cc} \; & \;\;\underset{u,v}{max}\;\left(u-d_{1}\right)\left(v-d_{2}\right)\\ \; & s.t.\left(u,v\right)\in S,\left(u,v\right)\geq \left(d_{1},d_{2}\right) \end{array}$$

The NB solution $\left(u^{*},v^{*}\right)$ maximizes the product of the players’ rewards relative to a fixed disagreement point.

### 4.2. DNB Solution for Multiple Players

This section describes the decentralized mechanism of the DNB controller for multiple players, more details of which were provided in [56]. In order to achieve maximum network performance, there is no intersection that needs to sacrifice its own performance. Each intersection will maximize its own performance individually. To calculate the reward function, which is the estimated sum of the queue lengths in each phase, vehicle speeds in each approach are needed. These are provided from the INTEGRATION microsimulator. In the simulations, if at time (*t*), the vehicle (*v*) speed ($s_{v}^{t}$) is less than the threshold speed ($s^{Th}$= 4.5(km/h)), the vehicle is assigned to the queue, and the current queue length ($q_{l}^{t}$) associated with the lane (*l*) is updated. If the vehicle speed exceeds ($s^{Th}$), the queue length is shortened by the number of vehicles leaving the queue. The mathematical equations for calculating the queue length in each lane are presented in Equations (30) and (31).

(30) $$q_{l}^{t}=\sum_{v\in v_{l}^{t}}q_{v}^{t}$$

(31) $$q_{v}^{t}=\left\{\begin{array}{cc} \;\;1 & \mathrm{if}\;s_{v}^{t-1}>s^{Th}\;\&\;s_{v}^{t}\leq s^{Th}\\ -1 & \mathrm{if}\;s_{v}^{t-1}\leq s^{Th}\;\&\;s_{v}^{t}>s^{Th}\\ \;\;0 & \left\{\begin{array}{c} \mathrm{if}\;s_{v}^{t-1}\leq s^{Th}\;\&\;s_{v}^{t}\leq s^{Th}\\ \mathrm{if}\;s_{v}^{t-1}>s^{Th}\;\&\;s_{v}^{t}>s^{Th} \end{array}\right. \end{array}\right.$$

After applying a certain action, the estimated queue length is calculated using Equation (32):

(32) $$Q_{P}\left(t+\Delta t\right)=\sum_{l\in P}q_{l}^{t}+Q_{inl}\Delta t-Q_{outl}\Delta t$$

where Δt is the update time interval, $q_{l}^{t}$ is the length of the current queue at time *t*, the estimated queue length for phase *P* after Δt is $Q_{P}\left(t+\Delta t\right)$, the flow rate of arrival is $Q_{inl}$, and the flow rate of departure is $Q_{outl}$. $Q_{outl}$ are measured at the downstream end of the links, and $Q_{inl}$ are measured at distances from the downstream end of the links equal to threat points over jam densities. The flows $Q_{inl}$ and $Q_{outl}$ can be measured using loop detectors or CCTV cameras.

The DNB solution can be computed as the vector that maximizes the product of the fixed threat point (*d*) relative to the player’s utility gains ($Q_{P}$), to reduce and equalize the queue lengths through the various phases. The threat point is the maximum queue length that each phase can accommodate (i.e., the maximum measurable length of the queue). The objective is to reduce the queue lengths across the different phases (*N*). The objective function can therefore be written accordingly:

(33) $$\begin{array}{cc} \; & \;\;\;\max_{\left(Q_{P1},\ldots ,Q_{PN}\right)}\prod_{i=1}^{N}\left(d_{i}-Q_{Pi}\right)\\ \; & s.t.\left(Q_{P1},.,Q_{PN}\right)\in S,\left(Q_{P1},.,Q_{PN}\right)\leq \left(d_{1},.,d_{N}\right) \end{array}$$

## 5. Testing on a Grid Network

This section presents the experimental setup, the experimental results, the statistical analysis of applying the proposed controllers on a grid network, and the summary findings.

### 5.1. Experimental Setup

**Figure 5:**
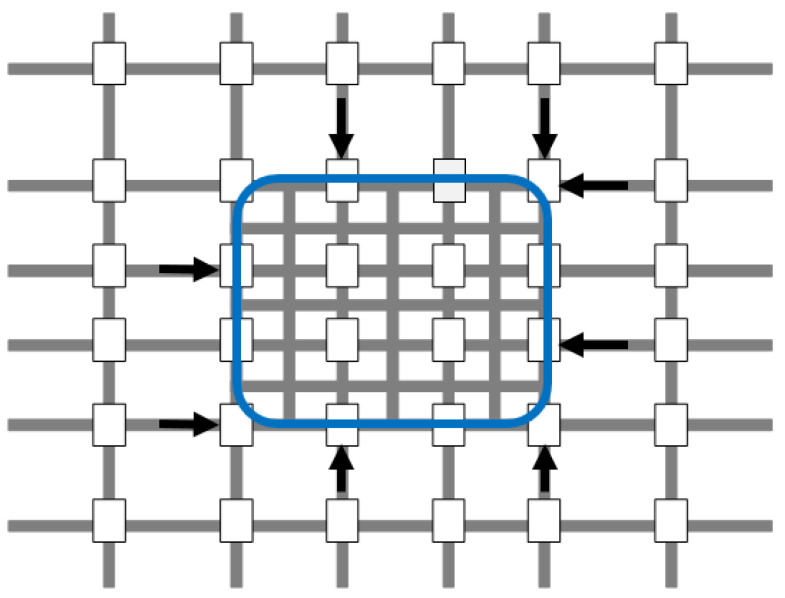
Protected network.

The test-bed network used in this study was modeled using INTEGRATION microscopic traffic simulation software. It is composed of 36 signalized intersections. The PN in Figure 5 is surrounded by the blue square, and the gates for this network are identified with the black arrows. The PN had 48 unidirectional links, where each link was 150m. In simulations, seven different traffic signal controllers (FP, PS, PSC, FPG, PSG, PSCG, DNB) were implemented and tested in the grid network. FP is a coordinated optimized fixed-time plan designed for relatively stable flows where the order and the duration of all phases remain fixed and do not adapt to traffic changes. PS is an adaptive centralized phase split controller that operates within a fixed cycle length. A common cycle length ensures the best traffic progression. PSC is a decentralized adaptive phase split and cycle length controller that dynamically optimizes phase splits and cycle lengths, without considering the coordination of the traffic signals. The advantage of such systems is that every traffic signal operates at its optimum traffic signal timings. Gating (G) is a feedback perimeter controller that is applied at the PN boundary traffic signals to prevent and/or delay the onset of traffic congestion. DNB is a decentralized traffic control system, which operates a flexible phasing sequence and free cycle length, through a Nash bargaining game-theoretic framework, to accommodate dynamic traffic demand changes. Decentralized systems are computationally less demanding, since only relevant information from the surrounding intersections/controllers is needed and maintained. In decentralized control systems’ robustness is guaranteed. The FP signal timings were optimized using the Webster method [57]. PS was optimized every 60 s, and PSC was optimized every 240 s. The minimum and maximum cycle lengths were 40 s and 120 s, respectively. Recent work showed that the Webster method overestimates the cycle length when the critical volume to capacity ratio exceeds 0.75 and proposed new procedures for estimating the optimum cycle length [58]. Future work will investigate the use of the modified formulae on the system’s performance.

To investigate the benefits obtained by using the developed DNB controller, we compared its operation with the operation of the gating controllers (i.e., FPG, PSG, and PSCG) and also to the operation of non-gating controllers (i.e., FP, PS, PSC). The operation of the feedback based gating control at the PN borders (i.e., FPG, PSG, and PSCG) was based on the NFD of the PN. The PN was congested when the average network density exceeded 48(veh/km) (i.e., $\bar{K}$; the density that corresponds to the maximum flow). Hence, gating controllers were activated at $0.85\;\bar{K}$ to avoid congestion. $A_{d}$ and $B_{d}$ were calibrated based on Equation (18). Subsequently, the $K_{P}$ and $K_{I}$ parameters were calculated using Equation (28). ${\bar{q}}_{in}$ is the steady state value of $q_{in}$ when *K* is around $\bar{K}$. The optimized parameters for the gating simulations were as follows: $\bar{K}=48\;\mathrm{veh}/\mathrm{km}$, ${\bar{q}}_{in}=4340\;\mathrm{veh}/h$, $A_{d}=0.782$, $B_{d}=0.00124$, $K_{P}=631$, $K_{I}=176$. For the DNB controller, the disagreement point was chosen as the number of vehicles that could be accommodated over a distance equal to half the link length. This ensures that queues do not spill back to the upstream intersections. The DNB update interval of 10 s was selected based on a sensitivity analysis of different update intervals.

### 5.2. Experimental Results

NFD curves show the controllers’ performance inside the PN, whereas the performance for the entire network (inside and outside the PN) was assessed using different measures of effectiveness (MOEs). More precisely, the average MOEs considering 20 different random seed simulations of each of the following was calculated: number of vehicle stops, travel time, total delay, vehicle fuel consumption, and vehicle emission levels (CO_2_).

**Figure 6:**
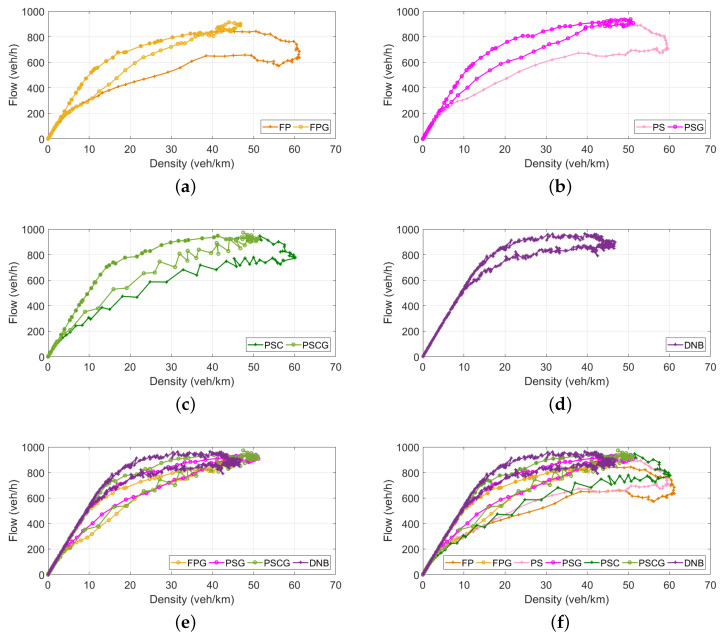
Average protected network fundamental diagram for 20 seeds: (**a**) fixed-time coordinated plan (FP) vs. FP with gating (FPG), (**b**) phase split controller (PS) vs. PSG, (**c**) phase split and cycle length controller (PSC) vs. PSCG, (**d**) DNB, (**e**) FPG vs. PSG vs. PSCG vs. DNB, and (**f**) all.

Figure 6 shows the NFD curves of the DNB controller along with all other controllers used in this study: FP, FPG, PS, PSG, PSC, PSCG. The results indicate that, applying the DNB controller, the PN never reached the congested regime (Figure 6d). Figure 6a–c shows that the gating controllers outperform non-gating controllers (the decreasing part of the NFD is eliminated by gating). Comparing the performance of the DNB controller with gating controllers (Figure 6e) shows that the DNB controller outperformed gating controllers with a higher flow ratio. Figure 6f compares the DNB with all other gating and non-gating controllers. It is clear from the figure that the DNB did not exceed the density at capacity (which was $\bar{K}=48\;\mathrm{veh}/\mathrm{km}$ in this study), and also, the DNB produced a higher vehicle throughput.

**Figure 7:**
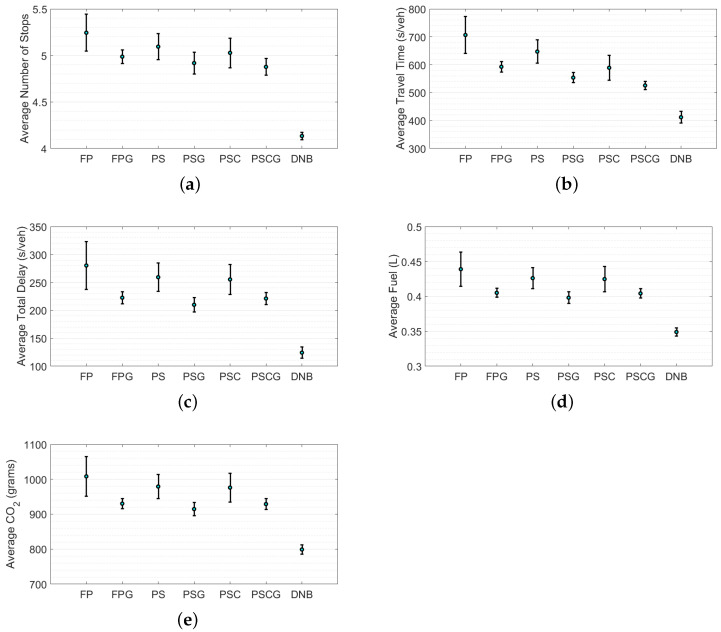
Average measures of effectiveness (MOEs) and standard deviations of 20 simulations at different seeds for the entire network: (**a**) average number of stops, (**b**) average travel time, (**c**) average total delay, (**d**) average fuel, and (**e**) average ${\mathrm{CO}}_{2}$.

To better evaluate the performance of the developed DNB controller over other (gating/non-gating) controllers, the average and standard deviations of each of the MOEs for the entire network (not only the PN) were calculated as shown in Figure 7. The results in Figure 7 show that the gating controllers (FPG, PSG, PSCG) outperformed the non-gating controllers (FP, PS, PSC). In addition, the figure shows that the DNB controller outperformed all the controllers with a significant reduction in both average values and standard deviations.

In addition, to further investigate the obtained improvements using the DNB controller, Table 2 shows the percentage improvements over the entire network using the DNB controller compared to other control strategies. The results show a significant reduction using the DNB controller over other controllers in average travel time from 21% to 41%, in total delay from 40% to 55%, and in CO_2_ emission and fuel consumption levels from 12% to 20%.

In summary, the results demonstrate that the addition of gating to state-of-the-art traffic signal controllers improves their performance, that the developed DNB controller does not need the assistance of a gating controller, and in fact, outperforms both (gating and non-gating) traffic signal controllers.

### 5.3. Statistical Analysis

To investigate the statistical significance of the findings, an analysis of variance (ANOVA) test was applied on all MOEs using the JMP software. The results showed that at least one controller had a statistically significant different mean. In addition, the Tukey test was applied to compare each controller to all other controllers. Table 3, Table 4 and Table 5 show the least squares mean (LSM) Tukey reports for the MOEs using the JMP software, where controllers not connected by the same letter were significantly different.

The Tukey test indicated that gating controllers (FPG, PSG, PSCG) were statistically different from non-gating controllers (FP, PS, PSC), respectively, and that the DNB controller was statistically significantly different in all MOEs compared to all other gating and non-gating traffic signal controllers.

### 5.4. Summary

In this section, the performance of the developed DNB controller is compared to state-of-the-art (FP, PS, PSC) and state-of-the-art gating/adaptive proportional-integral feedback (FPG, PSG, PSCG) traffic signal controllers. The proposed controllers’ performance was assessed using the INTEGRATION microscopic traffic assignment and simulation software. A total of 20 random seed simulations were conducted for each control strategy (FP, FPG, PS, PSG, PSC, PSCG, DNB). The NFDs of a PN for the different control strategies were investigated. The results showed that the DNB controller was able to prevent the formation of traffic congestion altogether. Comparing the performance of the DNB controller with other controllers revealed that the DNB controller outperformed the gating/adaptive and non-gating controllers. To better evaluate the DNB performance over other gating/non-gating controllers for the entire network (inside and outside the PN), the average and the standard deviations of each of the following measures of effectiveness (MOEs) were calculated: number of vehicle stops, vehicle travel time, total delay, vehicle fuel consumption, and vehicle emission levels. Results showed that gating controllers (FPG, PSG, PSCG) outperformed non-gating controllers (FP, PS, PSC) and that the DNB controller significantly reduced the average values and the standard deviations of all MOEs. The results showed that using the DNB controller versus other controllers led to significant reductions in average travel time from 21% to 41%, in total delay from 40% to 55%, and in emission levels (CO_2_) and fuel consumption from 12% to 20%. Analysis of variance and Tukey tests were conducted on all MOEs using JMP software. The DNB controller produced statistically significant improvements over other control strategies (gating and non-gating).

## 6. Conclusions and Recommendations for Further Work

In this paper, the performance of a developed decentralized traffic signal controller, which considers a flexible phasing sequence and free cycle length, based on a Nash bargaining game-theoretic framework (DNB) was compared to state-of-the-art adaptive and gating traffic signal control strategies, namely a centralized optimum fixed-time coordinated plan (FP), a centralized adaptive phase split controller (PS), and a decentralized adaptive phase split and cycle length controller (PSC). These controllers were combined with a state-of-the-art centralized gating proportional-integral feedback controller based on the network fundamental diagram (NFD). The gating was implemented at the PN border signals to limit vehicle entries to avoid or delay the onset of traffic congestion. The performances of the gating controllers (FPG, PSG, PSCG) and the non-gating controllers (FP, PS, PSC) were compared to the performance of the developed DNB traffic signal controller. The results show that the DNB controller is able to prevent the formation of traffic congestion and thus outperforms all other control strategies.

In addition to being less efficient than DNB, the gating control has other disadvantages. Gating control regulates the flow of traffic through the manipulation of the red indications at traffic signals upstream of a congested region, and the duration of gating depends on real-time measurements from the protected region. This may result in long queues and delays on the gated links. Gating at the border of a network may not be applicable if there is insufficient space to store the gated vehicles (queuing) or if there is an insufficient number of signalized intersections to gate the traffic. The network homogeneity condition should hold when using the NFD to derive control strategies. Gating control is centralized, where a central controller manages the input flow rate at the protected network gates. Alternatively, the DNB controller is decentralized, thus increasing the system’s scalability and stability, avoiding complex, centralized communication problems. Decentralized systems are usually economical to install and operate, as a reliable and direct communication network among the central computer and local controllers is not required.

In summary, the results revealed that gating controllers produce benefits for the state-of-the-art traffic control systems and that the developed DNB controller outperforms gating and non-gating traffic signal controllers. The results demonstrate the significant potential benefits of using the developed DNB controller over other state-of-the-art centralized and decentralized gating/adaptive traffic signal controllers.

For future work, the performance of the DNB controllers considering different levels of information (e.g., different levels of connected vehicle market penetration) and data noise should be investigated. Furthermore, further testing could consider the communication system and its impact on the performance of the various traffic signal control systems.

## Figures and Tables

**Table 1:** Two player matrix game.

		P_2_	
		**A_1_**	**A_2_**
**P_1_**	**A_1_**	$u_{1},v_{1}$	$u_{2},v_{2}$
	**A_2_**	$u_{3},v_{3}$	$u_{4},v_{4}$

**Table 2:** Average MOEs and the percent improvement using the DNB controller over the other controllers for the entire network.

	Controller	FP	FPG	PS	PSG	PSC	PSCG	DNB
MOE								
Average Number of Stops		5.244	4.987	5.094	4.918	5.028	4.877	4.134
Improvement %		21.16	17.10	18.85	15.94	17.78	15.23	
Average Travel time (s)		706.642	592.694	647.114	553.837	589.142	525.764	411.917
Improvement %		41.71	30.5	36.35	25.62	30.08	21.65	
Average Total Delay (s/veh)		280.335	222.699	259.410	210.037	255.413	221.227	124.298
Improvement %		55.66	44.19	52.08	40.82	51.33	43.81	
Average Fuel (L)		0.439	0.405	0.426	0.398	0.425	0.404	0.349
Improvement %		20.5	13.84	18.11	12.34	17.85	13.67	
Average CO_2_ (grams)		1008.209	930.148	979.152	914.559	976.064	928.839	798.699
Improvement %		20.78	14.13	18.43	12.67	18.17	14.01	

**Table 3:** Tukey test for total delay, fuel, and CO_2_.

Controller	Total Delay		Fuel		CO_2_	
	Class	LSM	Class	LSM	Class	LSM
FP	A	274.12	A	0.43	A	1000.43
PSC	A	257.18	A	0.42	A	978.29
PS	A	255.69	A	0.42	A	974.19
PSCG	B	222.12	B	0.40	B	930.06
FPG	B	222.06	B	0.40	B	929.09
PSG	B	208.18	B	0.39	B	911.79
DNB	C	124.62	C	0.34	C	799.07

**Table 4:** Tukey test for travel time.

Controller	Travel Time	
	Class	LSM
FP	A	697.68
PS	B	641.05
FPG	C	592.25
PSC	C	592.13
PSG	D	551.05
PSCG	D	527.19
DNB	E	412.30

**Table 5:** Tukey test for number of stops.

Controller	Number of Stops	
	Class	LSM
FP	A	5.24
PS	B	5.09
PSC	B C	5.02
FPG	B C D	4.98
PSG	C D	4.91
PSCG	D	4.87
DNB	E	4.13
